# Comparison of Medical Research Abstracts Written by Surgical Trainees and Senior Surgeons or Generated by Large Language Models

**DOI:** 10.1001/jamanetworkopen.2024.25373

**Published:** 2024-08-02

**Authors:** Alexis M. Holland, William R. Lorenz, Jack C. Cavanagh, Neil J. Smart, Sullivan A. Ayuso, Gregory T. Scarola, Kent W. Kercher, Lars N. Jorgensen, Jeffrey E. Janis, John P. Fischer, B. Todd Heniford

**Affiliations:** 1 Division of Gastrointestinal and Minimally Invasive Surgery, Department of Surgery, Atrium Health Carolinas Medical Center, Charlotte, North Carolina; 2 Department of Economics, Massachusetts Institute of Technology, Cambridge; 3 Division of Colorectal Surgery, Department of Surgery, Royal Devon & Exeter Hospital, Exeter, Devon, United Kingdom; 4 Department of Clinical Medicine, University of Copenhagen, Bispedjerg & Frederiksberg Hospital, Copenhagen, Denmark; 5 Division of Plastic and Reconstructive Surgery, The Ohio State University Wexner Medical Center, Columbus; 6 Division of Plastic Surgery, University of Pennsylvania Health System, Philadelphia

## Abstract

**Importance:**

Artificial intelligence (AI) has permeated academia, especially OpenAI Chat Generative Pretrained Transformer (ChatGPT), a large language model. However, little has been reported on its use in medical research.

**Objective:**

To assess a chatbot’s capability to generate and grade medical research abstracts.

**Design, Setting, and Participants:**

In this cross-sectional study, ChatGPT versions 3.5 and 4.0 (referred to as chatbot 1 and chatbot 2) were coached to generate 10 abstracts by providing background literature, prompts, analyzed data for each topic, and 10 previously presented, unassociated abstracts to serve as models. The study was conducted between August 2023 and February 2024 (including data analysis).

**Exposure:**

Abstract versions utilizing the same topic and data were written by a surgical trainee or a senior physician or generated by chatbot 1 and chatbot 2 for comparison. The 10 training abstracts were written by 8 surgical residents or fellows, edited by the same senior surgeon, at a high-volume hospital in the Southeastern US with an emphasis on outcomes-based research. Abstract comparison was then based on 10 abstracts written by 5 surgical trainees within the first 6 months of their research year, edited by the same senior author.

**Main Outcomes and Measures:**

The primary outcome measurements were the abstract grades using 10- and 20-point scales and ranks (first to fourth). Abstract versions by chatbot 1, chatbot 2, junior residents, and the senior author were compared and judged by blinded surgeon-reviewers as well as both chatbot models. Five academic attending surgeons from Denmark, the UK, and the US, with extensive experience in surgical organizations, research, and abstract evaluation served as reviewers.

**Results:**

Surgeon-reviewers were unable to differentiate between abstract versions. Each reviewer ranked an AI-generated version first at least once. Abstracts demonstrated no difference in their median (IQR) 10-point scores (resident, 7.0 [6.0-8.0]; senior author, 7.0 [6.0-8.0]; chatbot 1, 7.0 [6.0-8.0]; chatbot 2, 7.0 [6.0-8.0]; *P* = .61), 20-point scores (resident, 14.0 [12.0-7.0]; senior author, 15.0 [13.0-17.0]; chatbot 1, 14.0 [12.0-16.0]; chatbot 2, 14.0 [13.0-16.0]; *P* = .50), or rank (resident, 3.0 [1.0-4.0]; senior author, 2.0 [1.0-4.0]; chatbot 1, 3.0 [2.0-4.0]; chatbot 2, 2.0 [1.0-3.0]; *P* = .14). The abstract grades given by chatbot 1 were comparable to the surgeon-reviewers’ grades. However, chatbot 2 graded more favorably than the surgeon-reviewers and chatbot 1. Median (IQR) chatbot 2-reviewer grades were higher than surgeon-reviewer grades of all 4 abstract versions (resident, 14.0 [12.0-17.0] vs 16.9 [16.0-17.5]; *P* = .02; senior author, 15.0 [13.0-17.0] vs 17.0 [16.5-18.0]; *P* = .03; chatbot 1, 14.0 [12.0-16.0] vs 17.8 [17.5-18.5]; *P* = .002; chatbot 2, 14.0 [13.0-16.0] vs 16.8 [14.5-18.0]; *P* = .04). When comparing the grades of the 2 chatbots, chatbot 2 gave higher median (IQR) grades for abstracts than chatbot 1 (resident, 14.0 [13.0-15.0] vs 16.9 [16.0-17.5]; *P* = .003; senior author, 13.5 [13.0-15.5] vs 17.0 [16.5-18.0]; *P* = .004; chatbot 1, 14.5 [13.0-15.0] vs 17.8 [17.5-18.5]; *P* = .003; chatbot 2, 14.0 [13.0-15.0] vs 16.8 [14.5-18.0]; *P* = .01).

**Conclusions and Relevance:**

In this cross-sectional study, trained chatbots generated convincing medical abstracts, undifferentiable from resident or senior author drafts. Chatbot 1 graded abstracts similarly to surgeon-reviewers, while chatbot 2 was less stringent. These findings may assist surgeon-scientists in successfully implementing AI in medical research.

## Key Points

**Question:**

Can large language models generate convincing medical research abstracts?

**Findings:**

In this cross-sectional study comparing 10 medical abstracts written by surgical trainees and senior surgeons or generated by large language models, blinded expert surgeon-reviewers were asked to grade and rank these abstracts. There was no statistical difference in the grades or ranks of abstracts generated by the language model when compared with abstracts written by surgical trainees or senior surgeons.

**Meaning:**

These findings suggest that when appropriately trained with background literature, abstract formatting, primary research data, and a thorough prompt, chatbots can generate medical research abstracts that are difficult to distinguish from surgeon-scientist–written abstracts.

## Teaser

This cross-sectional study compares medical research abstracts written by surgical trainees or senior surgeons vs abstracts generated by artificial intelligence.

## Introduction

The introduction of artificial intelligence (AI) into the medical field has been both a promising and polarizing venture. Particularly, OpenAI Chat Generative Pretrained Transformer (ChatGPT; versions 3.5 and 4.0) is a new large language model, or chatbot, that has been trained from massive datasets to respond to prompts with sophisticated human-like answers.^1,2^ Medical professionals agree that these large language models have opened the door for new possibilities in medicine but also Pandora’s box. Arguments can be made for the benefit of AI in scientific research as well as for conflicts associated with AI in medicine.

The most common controversies associated with chatbots are the encroachment of plagiarism, biased training data, lack of creativity, and the spread of misinformation.^3^ Many surgeon-scientists worry that chatbots pull from sources that cannot be given proper credit, leading to plagiarism and copyright infringement.^4,5^ Although chatbots are trained on a plethora of information, there is little transparency in the data’s origin.^1,6,7^ As new reporting guidelines^7,8,9^ recommend how to describe the role of AI in a project, publishers and editors grapple with the listing of chatbots as an author. Some argue that chatbots should not be listed as an author because they cannot take responsibility for what is written.^1,7,10,11,12^ The ability of chatbots to generate novel ideas or think critically has also been questioned.^4,13,14,15^ Of particular concern is the spread of misinformation.^4,10^ Chatbots are not trained exclusively on medical texts, so there can be blatant inaccuracies (ie, hallucinations) in some of the AI responses.^2,16,17,18^ Chatbots state this information with a false confidence that precludes inaccuracy unless scrutinized by a well-versed health care clinician.^18^ Whether chatbots are endorsed by the scientific community or not, patients will inevitably use them to answer medical questions, so physicians should be invested in how to best validate the knowledge they emit.^4,15,18^

As a counterargument to these concerns, AI has several beneficial applications to the field of health care.^1,4,7,18,19,20,21^ Chatbots have demonstrated the ability to translate text^4,11^ and be integrated into hospital electronic medical records.^21^ They have even passed the US Medical Licensing Examination steps 1 and 2, which are required by medical students to earn their degree.^22^ The role of chatbots in scientific writing is being explored^23,24,25^ with the goal of improving efficiency and productivity of surgeon-scientists.^4,6,10,14^ If chatbots can be trained to assist in generating text for publication, scientists can devote more time to the complex pursuits involved in research.^1,2,4^ The goal of our study was to train 2 chatbots to generate medical research abstracts and assess how these abstracts compared with resident- and senior author–written abstracts as reviewed by blinded, well-published surgeons in the field. Furthermore, we evaluated the ability of chatbots to grade and rank medical abstracts when taught with a rubric.

## Methods

This cross-sectional study was performed at a tertiary care center in the Southeastern US and was determined exempt from review and the requirement of informed consent by the Carolinas Medical Center institutional review board. All abstracts utilized were written about a study previously approved by the Carolinas Medical Center institutional review board. This report follows Strengthening the Reporting of Observational Studies in Epidemiology (STROBE) reporting guideline. The study was conducted between August 2023 and February 2024 (including data analysis).

### Chatbot Training

OpenAI ChatGPT (versions 3.5 and 4.0; hereafter referred to as chatbot 1 and chatbot 2) was trained to generate medical abstracts based on provided abstracts as examples. The research residents and senior attending physician identified 10 abstracts^26,27,28,29,30,31,32,33,34,35^ by our group from 2012 to 2022 that were presented at national meetings and published in surgical journals to serve as the training models. There was variation in the first author of each abstract, a junior trainee, but all studies had the same senior author (B.T.H.). These abstracts were inputted as examples of our group’s writing style to provide few-shot learning (training an existing model by providing it examples to work from) for chatbot 1 and chatbot 2. The chatbots were prompted to note the similarities between the abstracts and confirm that they had saved our writing style. See eAppendix 1 in Supplement 1 for exact prompts.

### Chatbot Testing and Writing

Ten additional abstracts^36,37,38,39,40,41,42,43,44,45^ were used to investigate the chatbots’ ability to generate scientific abstracts. These abstracts were written by 5 different trainees within the first 6 months of their research year at the same medical center between 2018 to 2023 to account for the novice period. Abstracts from the current year’s research residents and fellows were excluded. All abstracts had the same senior author as the training abstracts (B.T.H.) and were submitted and presented at a variety of national and international conferences. Finally, these abstracts could only be included if we had access to the initial draft and final submitted version, the statistically analyzed research data, and a literature review of information concerning the topic of the abstract.

Once the chatbots were trained, we asked that it generate a scientific abstract based on the information provided. For each of the 10 abstracts, the chatbots were given the introduction and discussion of 3 relevant publications.^46,47,48,49,50,51,52,53,54,55,56,57,58,59,60,61,62,63,64,65,66,67,68,69,70,71,72,73,74,75^ Text limitations prevented us from giving the chatbots the entire article. Next, we provided our prompt.^6,16^ Specifically, we told the chatbots to generate text in the style of a senior surgeon-scientist with over 20 years of experience, like our senior author (B.T.H.). The analyzed real-world research data from each study was then pasted into the chat box. Finally, using the background literature, its knowledge as an experienced surgeon, and the data analysis, we asked both chatbot 1 and chatbot 2 to generate a version of each abstract in the trained writing style and in the specified format that was required by each national conference. An example prompt is available in eAppendix 1 in Supplement 1.

### Abstract Comparison

Once chatbot 1 and chatbot 2 generated abstracts of each of the 10 studies, these were compared with the resident’s first unedited draft and the senior surgeon’s edited, submitted version of the same abstract. The 4 versions were deidentified and sent to 5 blinded surgeon-reviewers (J.E.J., L.N.J, J.P.F., N.J.S., and K.W.K.). The 5 surgeons come from academic practices in Denmark, the UK, and the US, and all have served as presidents or board members of international surgical organizations or editorial boards with extensive experience in research and abstract writing and grading. The reviewers were asked to independently score the 4 versions of the abstracts on a 10- and 20-point scale. The 10-point scale was based on a typical abstract rubric. The 20-point scale was based on the American Society of Plastic Surgeons, which entailed 4 categories: completeness, relevance, quality, and exposure (each worth 5 points). See eAppendix 2 in Supplement 1 for the rubrics. The reviewers were also asked to force rank the 4 abstract versions from first to fourth, with first being the best abstract and fourth being the worst, with no ties. They were asked to repeat these grading methods for all 10 abstracts for a total of 40 versions. Additionally, in a separate session, we tasked chatbot 1 and chatbot 2 with grading all 40 abstract versions. The chatbots were provided with the same instructions on a standard 10-point rubric with 10 being the best and a 20-point rubric broken into 4 categories: completeness, relevance, quality, and exposure. See eAppendix 3 in Supplement 1 for the prompt and rubric provided to the chatbots.

### Statistical Analysis

Standard descriptive and comparison statistics were performed on the abstract versions using SAS version 9.4 (SAS Institute). The Fisher exact test was applied to compare categorical variables, and Kruskal-Wallis was utilized to compare continuous variables. All *P* values were 2-sided, and statistical significance was set at *P* < .05. We hypothesized that the chatbots would generate similarly graded and ranked abstracts as those written by surgical trainees and senior surgeons.

## Results

### Descriptive Statistics

Each surgeon-reviewer ranked an AI-generated version of an abstract first at least once, and 1 reviewer ranked either the chatbot 1 or chatbot 2 version first every time. The surgeon-reviewers ranked the resident’s version first 14 of 50 times and last 14 of 50 times. They ranked the senior author’s version first 13 of 50 times and last 13 of 50 times. The chatbot 1 version was ranked first least often (7 of 50 times) and ranked last most often (16 of 50 times). The chatbot 2 version was ranked first most often (16 of 50 times) and was ranked last least often (7 of 50 times) (Figure).

**Figure.**
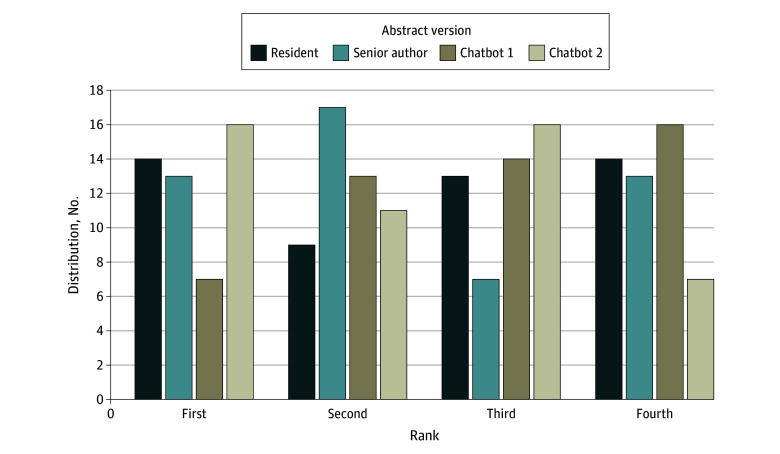
Distribution of Abstract Ranks by Surgeon-Reviewers The frequency that each abstract version was ranked (first, second, third, and fourth) by surgeon-reviewers. Chatbot 1 refers to Chat Generative Pretrained Transformer (GPT) version 3.5; chatbot 2, Chat-GPT version 4.0.

When the chatbots acted as the reviewer, chatbot 1 ranked its own version most favorably, ranking the resident’s version first only 1 of 10 times, the senior author’s version first 3 of 10 times, its own version first 5 of 10 times, and the chatbot 2 version first 1 of 10 times. Chatbot 1 ranked the resident’s version last 3 of 10 times, the senior author’s version last 2 of 10 times, its own version last 2 of 10 times, and the chatbot 2 version last 3 of 10 times. Contrastingly, chatbot 2 was more critical of its own abstracts. Chatbot 2 ranked the resident’s version first 2 of 10 times and the senior author’s version first 2 of 10 times, but it ranked the chatbot 1 version first 6 of 10 times and its own version first 0 of 10 times. Chatbot 2 never ranked chatbot 1 last and ranked itself last 4 of 10 times, the resident last 3 of 10 times, and senior author last 3 of 10 times.

When the frequency of ranks between surgeon-reviewer and chatbot-reviewer was compared, there was no statistical difference in the frequency that the resident or senior author’s abstracts were ranked; however, there was a statistical difference in how the chatbot 1 version and chatbot 2 version were ranked (Table 1). Both the surgeon-reviewers and chatbot-reviewers ranked the resident and senior author’s abstracts similarly, but they ranked chatbot 1 and chatbot 2 abstracts significantly differently. Surgeon-reviewers ranked chatbot 1 abstracts last frequently, while chatbot-reviewers did not. Surgeon-reviewers ranked chatbot 2 abstracts first frequently, while chatbot-reviewers ranked it worse.

**Table 1.** Frequency of Ranks by Surgeon-Reviewers Compared With Generative Language Model-Reviewers

Abstract version and rank^a^	Grader, No. (%)			*P* value^b^
	Surgeon (n = 50)	Chatbot 1 (n = 10)	Chatbot 2 (n = 10)	
Resident				
1	14 (28.0)	1 (10.0)	2 (20.0)	.67
2	9 (18.0)	4 (40.0)	1 (10.0)	
3	13 (26.0)	2 (20.0)	4 (40.0)	
4	14 (28.0)	3 (30.0)	3 (30.0)	
Senior author				
1	13 (26.0)	3 (30.0)	2 (20.0)	.76
2	17 (34.0)	2 (20.0)	2 (20.0)	
3	7 (14.0)	3 (30.0)	3 (30.0)	
4	13 (26.0)	2 (20.0)	3 (30.0)	
Chatbot 1				
1	7 (14.0)	5 (50.0)	6 (60.0)	.02
2	13 (26.0)	2 (20.0)	2 (20.0)	
3	14 (28.0)	1 (10.0)	2 (20.0)	
4	16 (32.0)	2 (20.0)	0	
Chatbot 2				
1	16 (32.0)	1 (10.0)	0	.04
2	11 (22.0)	2 (20.0)	5 (50.0)	
3	16 (32.0)	4 (40.0)	1 (10.0)	
4	7 (14.0)	3 (30.0)	4 (40.0)	

^a^ Abstracts were either written by a research resident within the first 6 months of their research year, were the final submitted version edited by a senior author, or were generated by chatbot 1 (Chat Generative Pretrained Transformer [GPT] version 3.5) or chatbot 2 (Chat-GPT version 4.0).

^b^ Statistical significance was *P* < .05.

### Chatbots as Abstract Generators

There was no statistical difference in the median (IQR) 10-point scores of the resident (7.0 [6.0-8.0]), senior author (7.0 [6.0-8.0]), chatbot 1 (7.0 [6.0-8.0]), or chatbot 2 (7.0 [6.0-8.0]) (*P* = .61). Again, on the 20-point scale, the surgeon-reviewers did not prefer the resident abstracts (median [IQR] score, 14.0 [12.0-17.0]) or senior author’s abstracts (median [IQR] score, 15.0 [13.0-17.0]) over the chatbot 1 (median [IQR] score, 14.0 [12.0-.16.0]) and chatbot 2 versions (median [IQR] score, 14.0 [13.0-16.0]) (*P* = .50). The reviewers’ median (IQR) rank did not differ significantly between abstract versions written by residents (3.0 [1.0-4.0]) or senior authors (2.0 [1.0-4.0]) and abstract versions generated by chatbot 1 (3.0 [2.0-4.0]) or chatbot 2 (2.0 [1.0-3.0]) (*P* = .14) (Table 2). When only comparing the reviews of chatbot 1 and chatbot 2, there was no statistical difference in the 10-point or 20-point scores, but the surgeon-reviewers statistically ranked chatbot 2 better (median [IQR] rank for chatbot 1, 3.0 [2.0-4.0] vs chatbot 2, 2.0 [1.0-3.0]; *P* = .02) (Table 3).

**Table 2.** Chatbots an Abstract Generator: Comparison of Grades by Surgeon-Reviewers^a^

Grading scale	Grade by surgeon reviewer, median (IQR)				*P* value^b^
	Resident	Senior author	Chatbot 1	Chatbot 2	
10-Point scale	7.0 (6.0-8.0)	7.0 (6.0-8.0)	7.0 (6.0-8.0)	7.0 (6.0-8.0)	.61
20-Point scale	14.0 (12.0-17.0)	15.0 (13.0-17.0)	14.0 (12.0-16.0)	14.0 (13.0-16.0)	.50
Rank	3.0 (1.0-4.0)	2.0 (1.0-4.0)	3.0 (2.0-4.0)	2.0 (1.0-3.0)	.14

^a^ Abstracts were either written by a research resident within the first 6 months of their research year, were the final submitted version edited by a senior author, or were generated by chatbot 1 (Chat Generative Pretrained Transformer [GPT] version 3.5) or chatbot 2 (Chat-GPT version 4.0).

^b^ Statistical significance was *P* < .05.

**Table 3.** Chatbots as an Abstract Generator: Comparison of Grades Subgroup Analysis: Chatbot 1 vs Chatbot 2^a^

Grading scale	Grade by surgeon reviewer, median (IQR)		*P* value^b^
	Chatbot 1	Chatbot 2	
10-Point scale	7.0 (6.0-8.0)	7.0 (6.0-8.0)	.41
20-Point scale	14.0 (12.0-16.0)	14.0 (13.0-16.0)	.41
Rank	3.0 (2.0-4.0)	2.0 (1.0-3.0)	.02

^a^ Abstracts were generated by chatbot 1 (Chat Generative Pretrained Transformer [GPT] version 3.5) or chatbot 2 (Chat-GPT version 4.0) and graded by 5 surgeon-reviewers.

^b^ Statistical significance was *P* < .05.

### Chatbots as a Grader

When comparing the surgeon-reviewers with chatbot 1 as a reviewer, there was no difference in their 10-point scores, 20-point scores, or ranks of any abstract version. Contrastingly, when comparing the surgeon-reviewers with chatbot 2 as a reviewer, there was a statistical difference in median grades and ranks. Particularly on the 20-point scale, chatbot 2 graded higher than the surgeon-grader for the resident’s abstract version (median [IQR] grade, 14.0 [12.0-17.0] vs 16.9 [16.0-17.5]; *P* = .02), the senior author’s abstract version (median [IQR] grade, 15.0 [13.0-17.0] vs 17.0 [16.5-18.0]; *P* = .03), the chatbot 1 abstract version (median [IQR] grade, 14.0 [12.0-16.0] vs 17.8 [17.5-18.5]; *P* = .002), and the chatbot 2 abstract version (median [IQR] grade, 14.0 [13.0-16.0] vs 16.8 [14.5-18.0]; *P* = .04). When the reviews by chatbot 1 and chatbot 2 were compared, again chatbot 2 gave higher median (IQR) grades for all 4 abstract versions on the 20-point scale (resident, 13.5 [13.0-15.0] vs 16.9 [16.0-17.5]; *P* = .003; senior author, 13.5 [13.0-15.5] vs 17.0 [16.5-18.0]; *P* = .004; chatbot 1, 14.5 [13.0-15.0] vs 17.8 [17.5-18.5]; *P* = .003; chatbot 2, 14.0 [13.0-15.0] vs 16.8 [14.5-18.0]; *P* = .01). See Table 4 for full analysis.

**Table 4.** Chatbots as a Grader: Comparison of Grades and Ranks Given by Surgeon-Reviewers vs Chatbot-Reviewers^a^

Abstract version	Grade, median (IQR)		*P* value^b^	Grade, median (IQR)		*P* value^b^	Grade, median (IQR)		*P* value^b^
	Surgeon-grader	Chatbot 1-grader		Surgeon-grader	Chatbot 2-grader		Chatbot 1-grader	Chatbot 2-grader	
10-Point scale									
Resident	7.0 (6.0-8.0)	7.0 (6.7-7.5)	.89	7.0 (6.0-8.0)	7.5 (7.5-7.8)	.24	7.0 (6.7-7.5)	7.5 (7.5-7.8)	.12
Senior author	7.0 (6.0-8.0)	7.3 (6.4-8.0)	.86	7.0 (6.0-8.0)	7.5 (7.5-7.8)	.13	7.3 (6.4-8.0)	7.5 (7.5-7.8)	.30
Chatbot 1	7.0 (6.0-8.0)	7.2 (6.5-7.8)	.10	7.0 (6.0-8.0)	8.2 (8.0-8.5)	.003	7.2 (6.5-7.8)	8.2 (8.0-8.5)	.02
Chatbot 2	7.0 (6.0-8.0)	7.3 (6.2-7.5)	.76	7.0 (6.0-8.0)	7.9 (7.0-8.0)	.14	7.3 (6.2-7.5)	7.9 (7.0-8.0)	.08
20-Point scale									
Resident	14.0 (12.0-17.0)	14.0 (13.0-15.0)	.79	14.0 (12.0-17.0)	16.9 (16.0-17.5)	.02	14.0 (13.0-15.0)	16.9 (16.0-17.5)	.003
Senior author	15.0 (13.0-17.0)	13.5 (13.0-15.5)	.28	15.0 (13.0-17.0)	17.0 (16.5-18.0)	.03	13.5 (13.0-15.5)	17.0 (16.5-18.0)	.004
Chatbot 1	14.0 (12.0-16.0)	14.5 (13.0-15.0)	.48	14.0 (12.0-16.0)	17.8 (17.5-18.5)	.002	14.5 (13.0-15.0)	17.8 (17.5-18.5)	.003
Chatbot 2	14.0 (13.0-16.0)	14.0 (13.0-15.0)	.79	14.0 (13.0-16.0)	16.8 (14.5-18.0)	.04	14.0 (13.0-15.0)	16.8 (14.5-18.0)	.01
Rank, quartile (range)									
Resident	3.0 (1.0-4.0)	2.5 (2.0-4.0)	.70	3.0 (1.0-4.0)	3.0 (2.0-4.0)	.54	2.5 (2.0-4.0)	3.0 (2.0-4.0)	.78
Senior author	2.0 (1.0-4.0)	2.5 (1.0-3.0)	>.99	2.0 (1.0-4.0)	3.0 (2.0-4.0)	.45	2.5 (1.0-3.0)	3.0 (2.0-4.0)	.56
Chatbot 1	3.0 (2.0-4.0)	1.5 (1.0-3.0)	.05	3.0 (2.0-4.0)	1.0 (1.0-2.0)	.002	1.5 (1.0-3.0)	1.0 (1.0-2.0)	.51
Chatbot 2	2.0 (1.0-3.0)	3.0 (2.0-4.0)	.10	2.0 (1.0-3.0)	2.5 (2.0-4.0)	.11	3.0 (2.0-4.0)	2.5 (2.0-4.0)	.94

^a^ Abstracts were either written by a research resident within the first 6 months of their research year, were the final submitted version edited by a senior author, or were generated by chatbot 1 (Chat Generative Pretrained Transformer [GPT] version 3.5) or chatbot 2 (Chat-GPT version 4.0).

^b^ Statistical significance was *P* < .05.

## Discussion

The first aim of this cross-sectional study was to evaluate if chatbots could generate scientific abstracts as well as a research resident or senior author. Based on 10- and 20-point scales, the abstracts were not differentiable. When force ranked, the chatbot 2 version was ranked first most frequently and the chatbot 1 version was ranked last most frequently. The second goal of this study was to assess how similarly chatbot- and surgeon-reviewers could grade abstracts. Chatbot 1 abstract grades were comparable to the surgeon-reviewers’ grades. However, chatbot 2 graded more favorably than the surgeon-reviewers and chatbot 1. Further observations were that the chatbots consistently utilized the provided results and did not hallucinate new data.

Although editors have worked quickly to regulate the implementation of AI in scientific writing, if it is permitted at all,^14^ AI continues to permeate all fields of medicine, academia, and research.^1,4^ The goal of this study was to evaluate if chatbots could generate and grade medical research abstracts. We found that, when trained using real-world data, chatbots could generate medical research abstracts in a manner that was not able to be differentiated from a human researcher. This is a promising and exciting observation, but further exploration should elucidate the ability of chatbots to consistently grade abstracts, given that the ability varied between chatbots 1 and 2 in our study. There are a variety of rubrics and scoring systems utilized in consideration for national meetings, but our findings indicate that a greater range point-system with defined categories is helpful to discern abstract quality. Abstract grading and consideration are time consuming, but the chatbots showed the potential to expedite this process and could help narrow down the number of abstracts human-reviewers need to read. Our group continues to explore the capability of chatbots as an abstract grader by more extensively training the AI model.

Despite successful implementation of AI in numerous areas of academia, like all new technologies, there is hesitancy to change.^15^ Chatbots gather information from unknown sources that cannot be directly cited, leading to controversy over plagiarism and copyright infringement.^4,5^ To combat this ethical dilemma, some investigators have asked chatbots to provide a list of references,^4,13^ but when cross-checked, the sources chatbots provided were sometimes falsified.^10,11^ In the medical field, where patient privacy is extremely important, there is a particular worry about the security of patient information shared with chatbots.^1,4^ Detractors have labeled chatbots a “stochastic parrot”^1^ that “threatens the trajectory”^13^ of modern medicine and scientific research. Some believe chatbots will stifle creativity, replace the learned ability of students to write papers, and degrade the sense of academic integrity.^14,15,76^ The counterargument is that learners still develop these writing skills, but in a nontraditional way, by editing chatbot output.^15^

Arguably, the most pertinent debate against chatbots is the spread of misinformation.^4,10^ The hallucinations^18^ produced by chatbots may present as fake statistics^77^ or inaccurate answers to medical questions. Emile et al^78^ assessed a chatbot’s ability to answer common questions about colon cancer, and Samaan et al^79^ reviewed the accuracy of a chatbot’s answers regarding bariatric surgery. Both found that the responses were mostly accurate, but there were certainly incorrect answers as well.^78,79^ Patients using chatbots may not be able to discern fact from fiction, so physicians, whether they support AI or not, should be invested in how their patients are using it.^4,15,18^

Despite these concerns, chatbots have potential in the medical community, including the potential to boost productivity in scientific writing. Chatbots can save researchers time by formatting papers specific to a journal,^1,4^ running statistics,^18^ and accelerating the publishing process, which alleviates pressure on surgeon-scientists.^4,6,10,14^ Chatbots can also be leveraged to reduce effort spent preparing a manuscript or grant by editing preexisting text, enhancing readability, and decreasing the number of rounds of feedback between authors.^4,10^ By increasing efficiency, some believe that chatbots can provide time to devote to more valuable pursuits.^1,2,4^ The ultimate goal of medical research is to advance knowledge and improve health for patients, so if we can employ AI^1,4^ to perform the routine tasks of research, we can spend more time on the creative aspects, complex questions, and critical thinking involved in research.

Prior studies have investigated the ability of chatbots to regenerate available medical research abstracts. Gao et al^77^ provided a chatbot with the title and journal name of previously published abstracts, while Levin et al^23^ provided the title and results section and asked it to regenerate the text. Gao et al^77^ found that human-reviewers correctly identified 68% of the chatbot-written abstracts and 86% of the human-written abstracts, but the chatbot versions were noted to be vague, making it easier to correctly distinguish them. Levin et al^23^ showed that AI-generated versions had fewer grammatical errors and more unique words than the scientist-written version, making these more difficult to distinguish.^24,25^

This study stands apart from prior work on AI-writing because the chatbots were provided with more than just a title and journal name.^77^ By training chatbots to generate text in our group’s writing style and inputting background, previously published studies, and statistically analyzed data for each abstract, we combatted the tendency for chatbots to hallucinate results. We suspect that as chatbots become more sophisticated, the potential to generate abstracts may surpass the ability of some researchers and may expand to generating full manuscripts.

One of the interesting observations we encountered while working with the chatbots was the variation between the chatbots 1 and 2. Both chatbot 1 and chatbot 2 were trained with data extending until September 2021, but chatbot 2 is considered the more advanced version^80^ and in our experience, had more independent thinking.^81^ When asking the chatbots to generate text, we used the same online session to provide consistency. Chatbot 1 was compliant and completed the tasks without needing redirection, but chatbot 2 had difficulty complying, required restarting new sessions, retraining each one, and several reminders of the prompt to finish writing all 10 abstracts. Although we intended to train the chatbots on more than 10 abstracts, often after the fifth abstract, chatbot 2 pushed back, stating that it did not need more abstracts to learn the writing style. We proceeded, however, in training the chatbots with 10 abstracts. Despite chatbot 2 being less compliant, blinded surgeons agreed that the chatbot 2 abstract versions were better and more consistent than the chatbot 1 versions. The chatbots followed directions on grading more easily, suggesting future promise in saving researchers and editors’ time.

Both advocates and skeptics mostly agree that chatbots will not replace surgeons as primary decision makers in the near future.^4,6,17,21^ AI has the potential to complement patient-clinician interactions and assist in medical research, but it will be difficult for AI to replace a surgeon’s judgement.^6,17,21^ Chatbots can serve as a helpful ally in medical abstract generating and grading, but at this point in its evolution, AI cannot perform independently. In the meantime, our goal is to leverage AI for the function of better research and ultimately better patient care.^4,14^ AI is permeating all facets of medicine, and as clinicians, we need to decide the best approach to incorporate it into our research and clinical space.

### Limitations

The primary limitation of this study was the small sample size of abstracts and reviewers. To combat this limitation, we intentionally chose surgeons who had extensive experience and represented different practice models and international backgrounds. Furthermore, this work is based on abdominal wall reconstruction abstracts and thus may not translate to other fields of medicine. There are also limitations of chatbots. The chatbots have a knowledge cutoff in September 2021 and do not have the ability to browse the internet for more recent context. Chatbots are dependent on the data and training they received, which could result in bias that they learned.^3,82^ The chatbots additionally have a token cutoff, or character limit, which may inhibit the quantity of training or prompting the model can learn at a time.^17^

## Conclusions

The findings of this cross-sectional study suggest that a chatbot can generate quality medical research abstracts when the user spends the time to train it, feed it background information, and supply it with analyzed data. The chatbots in this study also demonstrated the ability to grade abstracts, with chatbot 2 being less stringent than chatbot 1. The findings of this study serve as an example of successful and safe implementation of AI in scientific writing, which we hope is considered as editors and publishers continue to determine the regulation and acceptable role of AI.

## Notes

Supplement 1. **eAppendix 1.** Chat GPT Training and Writing Prompts **eAppendix 2.** 10-Point and 20-Point Scale Rubrics **eAppendix 3.** Chat GPT Grading Prompts

Supplement 2.
Data Sharing Statement
