# Supplementary material for: Comparison of Medical Research Abstracts Written by Surgical Trainees and Senior Surgeons or Generated by Large Language Models
Source: JAMA Netw Open. 2024 Aug 2;7(8):e2425373. doi: 10.1001/jamanetworkopen.2024.25373 (PMC11297395; doi:10.1001/jamanetworkopen.2024.25373)
Supplement: Supplement 1. — eAppendix 1. Chat GPT Training and Writing Prompts eAppendix 2. 10-Point and 20-Point Scale Rubrics eAppendix 3. Chat GPT Grading Prompts [file jamanetwopen-e2425373-s001.pdf]

Supplemental Online Content

Holland AM, Lorenz WR, Cavanagh JC. Comparison of medical research abstracts written by surgical trainees and senior surgeons or generated by large language models. *JAMA Netw Open*. 2024;7(8):e2425373. doi:10.1001/jamanetworkopen.2024.25373

Appendix 1. Chat GPT Training and Writing Prompts

Appendix 2. 10-Point and 20-Point Scale Rubrics

Appendix 3. Chat GPT Grading Prompts

This supplemental material has been provided by the authors to give readers additional information about their work.

© 2024 Holland AM et al. *JAMA Network Open.*

eAppendix 1. Chat GPT Training and Writing Prompts

"I'd like your help in creating scientific abstracts for our abdominal wall reconstruction research. Your first task will be to understand my writing style based on examples that I give you. After that, we'll create some content. To start, please say GO AHEAD, and I will paste 10 total examples of my writing. Keep saying GO AHEAD and I will paste new examples. When I am done, I will say FINISHED. At this stage, please do not do anything except confirm that you have saved the writing style."

"Next, I am going to ask you to write a scientific abstract based on a prompt and data I give you. Please write the abstract as though you are a surgeon with over 20 years of experience and an extensive research background. I will provide you with pieces of background information from 3 relevant papers. When writing the abstract, you should mostly pull from this content, but if you need more information please look elsewhere. For the background of this abstract, you should pull from your knowledge as an experienced surgeon in addition to the attached content of the relevant papers. Please say GO AHEAD when you are ready and GO AHEAD after each publication. I will say FINISHED when we are ready to write."

"Thank you! Using "our writing style" and the background information you just learned from the 3 prior publications, please write a scientific abstract with a title and these sections: background, methods, results, conclusions, in paragraph form."

"Please write an abstract about the outcomes of coated versus uncoated polypropylene mesh in open ventral abdominal hernia repairs. Barrier coated mesh aims to reduce adhesion formation between the bowel and mesh, but there is not much data about the risk of infection with rapidly absorbable coatings. The primary outcome is mesh infection and wound infection. Please write the abstract between 2500-3000 characters, including spaces but not including the title."

© 2024 Holland AM et al. *JAMA Network Open.*

eAppendix 2. 10-Point and 20-Point Scale Rubrics

Use a basic 10-point scale to grade each abstract with 1 being the worst and 10 being the best.

For the 20-point scale, use the 4 categories below and award up to 5 points for each of the categories.

4 categories

1. Completion quality for submission – includes a concise purpose/significance, originality, methods, number of cases with follow-up data, summary of results, understandable conclusions
2. Relevance of the data to the meeting's field of medicine
3. Quality of abstract – well thought out and articulated
4. Exposure factor – innovative or has it already had significant exposure in the field

Please grade each category out of 5 for a total of 20 points

1- Not acceptable
2- Acceptable
3- Good
4- Very good
5- Excellent

eAppendix 3. Chat GPT Grading Prompts

"You are a surgeon with 20 years of experience in clinical research; based on a brief rubric and your experience you grade abstracts for inclusion in a surgical research conference.

I am going to provide you with two brief rubrics first. Please review the rubrics and when you are ready to grade the abstracts, say GO AHEAD, and I will then send four research abstracts submitted to the 2023 academic surgical conference.

Rubric 1: Grade the abstract on a scale of 1-10. Best abstracts: 8.1-10.0. Good abstract, qualifies for oral presentation: 6.1-8.0. Fair abstract, qualifies for a poster presentation or short "quick shot" oral presentation: 4.1-6.0. Poor abstract, qualifies for a poster presentation: 2.1-4.0. Dangerous abstract: 0.0-2.0

Rubric 2: Grade the abstract on a scale of 1-20. There are four categories, each with a total of 5 points. Please combine the scores for all four categories to get the final score out of 20. The first category is "completion quality of submission," meaning it is concise, original, includes a methods section, summarizes the results, and has understandable conclusions. The second category is "relevance to the field of medicine," meaning the abstract discusses an important surgical topic. The third category is "quality of abstract," meaning the abstract is well articulated and eloquently written. The fourth category is "exposure factor," meaning the topic is interesting or innovative in the field of surgery.

Finally, please rank the four abstracts in order of best to worst, 1st being the best and 4th being the worst.

You are going to grade each abstract twice and rank each abstract once, so that each abstract will have a total of three scores. Please grade each of the four abstracts according to the brief rubrics above. Please provide one final score out of 10 and a final score out of 20 for each abstract. Please organize the three scores in a spreadsheet for each abstract.

Do you understand? If you understand and have reviewed the rubrics, please say GO AHEAD and I will send the abstracts."

© 2024 Holland AM et al. *JAMA Network Open.*
