# Supplementary material for: Comparison of Medical Research Abstracts Written by Surgical Trainees and Senior Surgeons or Generated by Large Language Models
Source: JAMA Netw Open. 2024 Aug 2;7(8):e2425373. doi: 10.1001/jamanetworkopen.2024.25373 (PMC11297395; doi:10.1001/jamanetworkopen.2024.25373)
Supplement: Supplement 2. — Data Sharing Statement [file jamanetwopen-e2425373-s002.pdf]

Data Sharing Statement

Holland. Comparison of Medical Research Abstracts Written by Surgical Trainees and Senior Surgeons or Generated by Large Language Models. *JAMA Netw Open*. Published August 02, 2024. doi:10.1001/jamanetworkopen.2024.25373

Data
Data available: No
